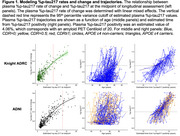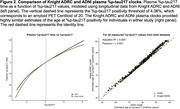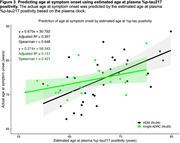# Predicting onset of Alzheimer disease symptoms with a plasma %p‐tau217 clock

**DOI:** 10.1002/alz70856_099674

**Published:** 2025-12-24

**Authors:** Kellen K. Petersen, Marta Milà‐Alomà, Duygu Tosun, Yan Li, Benjamin A. Saef, Leslie M. Shaw, Jeffrey L. Dage, Henrik Zetterberg, Lei Du‐Cuny, Chengjie Xiong, Nicolas R. Barthélemy, Randall J. Bateman, David M. Holtzman, John C. Morris, Anthony W. Bannon, William Z. Potter, Suzanne E. Schindler

**Affiliations:** ^1^ Department of Neurology, Washington University School of Medicine, St. Louis, MO, USA; ^2^ Department of Radiology and Biomedical Imaging, University of California, San Francisco, San Francisco, CA, USA; ^3^ Department of Veterans Affairs Medical Center, Northern California Institute for Research and Education (NCIRE), San Francisco, CA, USA; ^4^ Washington University in St. Louis, School of Medicine, St. Louis, MO, USA; ^5^ Washington University in St. Louis School of Medicine, St. Louis, MO, USA; ^6^ Perelman School of Medicine, University of Pennsylvania, Philadelphia, PA, USA; ^7^ Stark Neurosciences Research Institute, Indiana University School of Medicine, Indiana, IN, USA; ^8^ Department of Neurology, Indiana University School of Medicine, Indianopolis, IN, USA; ^9^ Institute of Neuroscience and Physiology, Sahlgrenska Academy at the University of Gothenburg, Göteborg, Sweden; ^10^ Hong Kong Center for Neurodegenerative Diseases, Clear Water Bay, Hong Kong, China; ^11^ University College London, Dementia Research Institute, London, United Kingdom; ^12^ Wisconsin Alzheimer's Disease Research Center, University of Wisconsin School of Medicine and Public Health, University of Wisconsin‐Madison, Madison, WI, USA; ^13^ Clinical Neurochemistry Laboratory, Sahlgrenska University Hospital, Mölndal, Sweden; ^14^ Department of Neurodegenerative Disease and UK Dementia Research Institute, UCL Institute of Neurology, Queen Square, London, United Kingdom; ^15^ AbbVie Deutschland GmbH & Co KG, Ludwigshafen, BW, Germany; ^16^ Washington University School of Medicine in St. Louis, St. Louis, MO, USA; ^17^ Department of Neurology, Washington University in St. Louis School of Medicine, St. Louis, MO, USA; ^18^ The Tracy Family SILQ Center, St. Louis, MO, USA; ^19^ Hope Center for Neurological Disorders, Washington University in St. Louis, St. Louis, MO, USA; ^20^ Knight Alzheimer Disease Research Center, St. Louis, MO, USA; ^21^ Knight Alzheimer Disease Research Center, Washington University School of Medicine, St. Louis, MO, USA; ^22^ Washington University in St. Louis, St. Louis, MO, USA; ^23^ AbbVie Inc., North Chicago, IL, USA; ^24^ Highly qualified expert, Philadelphia, PA, USA

## Abstract

**Background:**

Time proxies based on blood biomarkers may facilitate understanding of the timing of biomarker change and symptom onset in Alzheimer's disease (AD) and could potentially enable biological staging of AD. Compared to CSF biomarkers or brain imaging, blood biomarkers are more accessible and less burdensome, making a “plasma clock” a practical and useful tool.

**Method:**

Longitudinal plasma %p‐tau217 (*p*‐tau217 concentration divided by np‐tau217 concentration x 100) measurements from individuals enrolled in the Knight ADRC (*n* = 420) or ADNI (*n* = 392) cohorts were analyzed to develop plasma clocks for each cohort. The %p‐tau217 values were transformed into a time scale by integrating the inverse of the modeled rate of change. A %p‐tau217 value of 4.06%, which corresponds to amyloid PET Centiloid=20, was considered the threshold for positivity and set as time zero. The estimated age at %p‐tau217 positivity was then used to predict symptom onset, defined as a change in Clinical Dementia Rating® (CDR) from CDR=0 to CDR>0.

**Result:**

The rate of change for plasma %p‐tau217 was relatively consistent between 0.65‐8.16% (Figure 1) and was used to construct independent plasma %p‐tau217 clocks for the Knight ADRC and ADNI cohorts (Figure 2). For individuals with plasma %p‐tau217 measured before and after positivity (4.06%), the estimated age at positivity based on the clock was strongly correlated with the actual age at conversion (Knight ADRC: R2=0.646, r=0.807; ADNI: R2=0.790, r=0.891). The Knight ADRC and ADNI plasma clocks provided highly similar estimates of the age at %p‐tau217 positivity for individuals in either study (adjusted R^2^=0.993, Pearson r=0.997, Figure 2). The estimated age at plasma %p‐tau217 positivity predicted the age at symptom onset (Figure 3) in the Knight ADRC (Spearman r=0.421, adjusted R^2^=0.151) and ADNI cohorts (Spearman r=0.648, adjusted R^2^=0.397).

**Conclusion:**

Two independently constructed %p‐tau217 plasma clocks had strong correlations with actual time measures and were consistent across the two cohorts. Additionally, the age at %p‐tau217 positivity predicted symptom onset. Overall, these findings suggest that plasma %p‐tau217 may be useful in predicting symptom onset and in biological staging of AD.